# A new therapeutic approach for bone metastasis in colorectal cancer: intratumoral melittin

**DOI:** 10.1590/1678-9199-JVATITD-2021-0067

**Published:** 2022-03-14

**Authors:** Mackson Martins Rocha, Isabela Dariva, Gabriela Comelli Zornoff, Giovanna Sanches De Laurentis, Giulia Carli Mendes, Maycon Giovani Santana, Guilherme Chohfi de Miguel, Rui Seabra Ferreira, Juliana Mozer Sciani, Denise Gonçalves Priolli

**Affiliations:** 1 Multidisciplinary Laboratory, Postgraduate Program in Health Sciences, São Francisco University, Bragança Paulista, SP, Brazil.; 2 Medical School, São Francisco University, Bragança Paulista, SP, Brazil.; 3 Nursing School, São Francisco University, Bragança Paulista, SP, Brazil.; 4 Center for the Studies of Venoms and Venomous Animals (CEVAP), São Paulo State University (UNESP), Botucatu, SP, Brazil.

**Keywords:** Bone metastasis, Melittin, Intratumoral injection, Colorectal neoplasms, Bee venom

## Abstract

**Background:**

Melittin has shown antiproliferative effects on tumor cells. Therefore, it comprises a valuable compound for studies on cancer treatment. To the best of our knowledge, no studies have reported melittin effects on bone metastasis. Herein, we propose an approach based on intrametastatic melittin injection to treat bone metastases in colorectal cancer.

**Methods:**

Following the characterization of melittin and antiproliferative tests *in vitro*, a single dose was injected through intrametastatic route into the mouse bone metastasis model. Following treatment, metastasis growth was evaluated.

**Results:**

A single dose of melittin was able to inhibit metastasis growth. Histological analysis showed necrosis and inflammatory processes in melittin-treated metastasis. Except by mild weight loss, no other systemic effects were observed.

**Conclusion:**

Our data suggest that melittin might be a promising agent for the future development of treatment strategies aiming to reduce the bone metastasis skeletal-related impact in colorectal cancer patients with bone metastasis.

## Background

According to a cancer report published by the World Health Organization (WHO) in 2020, more than 18 million cancer cases were registered worldwide in 2018. The WHO also predicts that the world's total number of cases in 2020 will be around 29 million worldwide [1]. 

Colorectal cancer (CRC) remains the third leading cause of cancer-related mortality globally, despite all efforts in screening, early detection, and recent advances in treatment modalities [2]. Approximately 22% of CRCs are metastatic at initial diagnosis, and 70% of patients in the disease course will develop it. Given the heterogeneity of metastatic tumors, predicting metastatic survival outcomes remains challenging. Patients with metastatic CRC have a poor prognosis in general, with a relative 5‐year survival rate of 14%, compared to 71% and 90% in those with regional and localized CRC, respectively [3]. Moreover, the therapy is focused on the patients' quality of life increase by reducing bone metastasis skeletal-related impact [4]. WHO defines quality of life as “an individual's perception of their position in life in the context of the culture and value systems in which they live and in relation to their goals, expectations, standards and concerns*”* [5].

Skeletal-related events (SREs) in bone metastases "represent a difficult to treat clinical scenario due to pain, increased fracture risk, decreased quality of life, and diminished overall survival outcomes" [6]. Currently, the medical management of SREs is based on local or systemic approaches [7,8]. There are several studies regarding this pathology; however, there are no efficient clinical methods for its cure or prevention [9-13]. 

Currently, multi-bone metastasis from solid tumors is treated with bone-targeted agents. Although expansive, the therapeutic implications remain limited [11,13], and there is no guarantee of efficacy and pain prevention [11]. Conventional systemic treatments are not very effective due to their low bone distribution because of bone vascularization. 

Recent progress in metastasis research has vastly expanded our understanding of the cellular and molecular levels [14-17]. Since most studies have focused on liver metastasis, site-specific treatments, such as bone metastasis, have been poorly studied [14,16,17,18]. Thus, novel less expensive therapeutics to improve survival, decrease fracture risk, and alleviate pain in patients with metastatic bone cancers are needed. One potential source for such substances might be bee venom.

Many studies described bee venom components' biological activities [19-21]. They launched preclinical trials to improve its constituents as the next generation against neurodegenerative diseases, inflammatory diseases, and drugs for cancer [22].

Melittin from *Apis mellifera*, bee venom most abundant component, accounts for 40%-60% of its total dry weight. It is a 26 amino acid amphipathic peptide, in a monomer and a tetrameric architecture, spontaneously formed in an aqueous environment [23-25]. Moreover, this peptide is known by its cytolytic activity - action on the cell membrane through the pore forming and transmembrane protein helices [26].

Due to its cytolytic effect, melittin has been tested in tumor cells, and has been shown to selectively induce cell death in cancer cells [27-34]. 

Particularly on colon cancer cells, bee venom demonstrated apoptotic effects by activating death receptors and inhibiting nuclear factor kappa B [33,35,36] without affecting fetal colon epithelial cells and colon epithelial non-tumor cells [36]. Moreover, melittin reduced the growth of human colorectal tumor cells (CT26 and LS174T) by inhibition of protein translation and synthesis. The cytotoxicity was assessed by changing the cell membrane in COLO205 and HCT-15 colorectal tumor cells at high concentrations [37]. In addition, the cytolytic action caused phospholipid bilayer rupture and pore formation, increased cell membrane permeability, and activated several intracellular pathways that induced apoptosis [38]. After membrane damage, melittin mechanism is also related to necrosis in colorectal tumor cells [35] and showed elevated redox potential [39-41]. 

Taking this into account, melittin might be a promising agent for strategies aiming at reducing bone metastasis SREs in CRC patients. Nevertheless, one of the obstacles to melittin treatment is its high toxicity, which can induce severe complications such as hemolysis, coagulopathy, thrombocytopenia, rhabdomyolysis, and liver dysfunction by systemic administration [26,35,42]. Melittin conjugates and derivates have been described in most recent cancer studies [27-34,37,43] eradicating 100% of the tumor cells, including colon cancer cells, without adverse side effects [35,41]. Therefore, in the present study we propose a new approach based on melittin intrametastatic therapy to treat bone metastasis in CRC.

## Methods

### Melittin identification

Melittin was purified from *Apis mellifera* venom collected from apiaries in the region of Botucatu, Brazil (22° 53' 09" S 48° 26' 42" O). Purification was conducted according to a previous report [26]. 

The purity and identity were confirmed by mass spectrometry using a Q-Exactive Plus (Thermo Fisher Scientific, Waltham, Massachusetts, USA). The peptide was inserted into a C18 column (EASY-Spray™ LC Columns, Thermo Fisher Scientific, Waltham, Massachusetts, USA) coupled to a liquid chromatography binary system (EASY-nLC 1,200, Thermo Scientific, Waltham, Massachusetts, USA) and eluted with a gradient of 5%-80% of solvent A (water containing 0.1% formic acid) and B (90% acetonitrile containing 0.1% formic acid) with a constant flow of 100 nL/min. The eluted was automatically inserted in the mass spectrometer, operating in positive mode, in MS mode of a full scan of 300-1500 m/z [26].

### Cell culture

Human CRC (HT-29) was purchased from the European Collection of Authenticated Cell Cultures. It was thawed and propagated in 25 cm^3^ flasks at 37 °C in a 5% CO_2_ humidified chamber (HeraCELL 150) in Dulbecco's modified Eagle's medium (DMEM; Sigma D-5648, São Paulo, Brazil) supplemented with 100 mM sodium pyruvate (Gibco, Thermo Fisher Scientific, Waltham, Massachusetts, USA), 10% fetal bovine serum (FBS) (Gibco, Fisher Scientific, Waltham, Massachusetts, USA), and 1% antibiotics (100 U/mL penicillin and 10 mg/mL streptomycin, Gibco). Cells were detached using 0.25% trypsin-EDTA (Gibco, Fisher Scientific, Waltham, Massachusetts, USA) at 37 °C for 3 min. DMEM plus 10% FBS was used to block the trypsin. The cell pellet was transferred to a new 75 cm^3^ flask containing 10 mL DMEM. The culture medium was changed every 24 h. Cell viability was evaluated in the Neubauer chamber using Trypan Blue.

### MTT cytotoxicity assays

To check the viability, the culture fluid is removed and 5 × 10^5^cells per well were treated with melittin (0.0625, 0.125, 0.25, 0.5, 1.0, 2.0, 35 and 70 µM). After 48 h of incubation, MTT (3-(4,5-dimethylthiazol-2-yl)-2,5-diphenyltetrazolium bromide) 0.5 mg/mL was added to each well and incubated at 37 °C for 4 h to determine the number of living cells [44]. The formed formazan crystals were dissolved in DMSO and shaken for 10 min to dissolve the crystals. Then, the optical density was detected by a microplate reader at a wavelength of 540 nM (EPOCH, BioTech Instrument Inc., Winooski, VT, USA). Each experiment was repeated three times (triplicates).

### Animal model for bone metastasis in colorectal cancer

All applicable international, national, and institutional guidelines for the care and use of animals were followed. This research study followed the National Council of Animal Experience Control, the Guide for the Care and Use of Laboratory Animals and ARRIVE guidelines. All procedures and efforts were made to minimize suffering and performed following ethical standards. The animal study was reviewed and approved by the Ethics Experimentation Animal Committee from the São Francisco University (# 006.03.19), Brazil. 

Six-week-old Balb/c-nu male mice (n = 9), weighing 23,76 ± 3,11 g, from Charles River Laboratories International Inc. (Wilmington, USA) were housed in individual ventilated racks. All animals were kept under controlled light conditions (12 h light/12 dark cycles), temperature (23 ± 1 °C), humidity (40%-60%), water, and feed ad libitum.

The procedures were performed in a laminar flow with rigorous asepsis and antisepsis techniques [45]. On day 0, to perform the cell inoculation, mice were anesthetized with xylazine hydrochloride 2% plus ketamine, diluted 1:2 (0.3 mL/20 g), and administered via intramuscular injection. Briefly, cells were suspended in 40 μL of saline. A percutaneous 45° puncture was performed. Bone scarification was performed with the bevel of the hypodermic needle carefully to avoid transfixing the skullcap. Then, without needle exchange, cells (4 × 10^6^ cells) were injected over the periosteal in the parietal region using a 1 mL syringe and 36G caliber hypodermic needle [46].

After xenograft, the animals were monitored daily. Metastasis was verified and measured every day. In case of signs, the animal is euthanized immediately. Metal calipers checked the volume. The growth curves were determined using the formula: Volume = L × S^2^/2, where "S" is the smallest diameter measured and "L" is the largest diameter measured.

### Experimental procedure

After the tumor reached 100 mm^3^ (by the 19^th^ day after xenograft), the animals were randomized into groups:

Control (n = 3) - Bone CRC metastasis, untreated.

Melittin (n = 6) - Metastasis treated with melittin.

After anesthesia, 1,5 mg/kg single dose of melittin was injected in the metastasis with a sterile syringe, as deep as possible, without trespassing the tumor. Without removing the needle, 50 µL of solution was applied slowly for a homogenous distribution into the metastasis, equally distributed (10 µL) in the center and cardinal points. After the administration, the needle remained in the metastasis for 20 seconds before being slowly removed to avoid extravasation.

### Animal follow up

Signals of suffering and toxicity were carefully observed daily. The following general behaviors were observed: low activity, appetite loss, personality changes, immobility, social isolation, urine and feces consistency changes, lack of personal hygiene, and self-mutilation. Specific to mice, weight loss, dehydration, piloerection, and screams when touched were evaluated.

### Histopathological analysis

After the anesthetic procedure by the parenteral anesthetic drug overdose, the animals were euthanized. The tumor was then resected entirely for anatomopathological analysis. The external surface of the sample was inspected. The opened and cleaned specimens were immersed in formalin overnight for fixation and embedded in paraffin. Metastasis was sliced to assess bone invasion. Tumor 3 µm sections were stained with hematoxylin-eosin and subjected to optical microscopy. Metastasis diagnosis and histological features were determined.

Moreover, immediately after euthanized, liver, kidneys, and heart were removed from the animals for histopathological analysis, which was conducted with the same method described above.

### Statistical analysis

The statistical power was previously determined and showed that the "n" is consistent with the hypothesis [47]. A p-value ≤ 0.05 was considered significant to reject the null hypothesis using the following models: descriptive statistics, measures of central tendency, normality test, and Mann-Whitney test. SPSS for Windows version 21.0 was used for all analyses. Results are presented as mean and standard deviation (SD).

## Results

**Figure 1.**
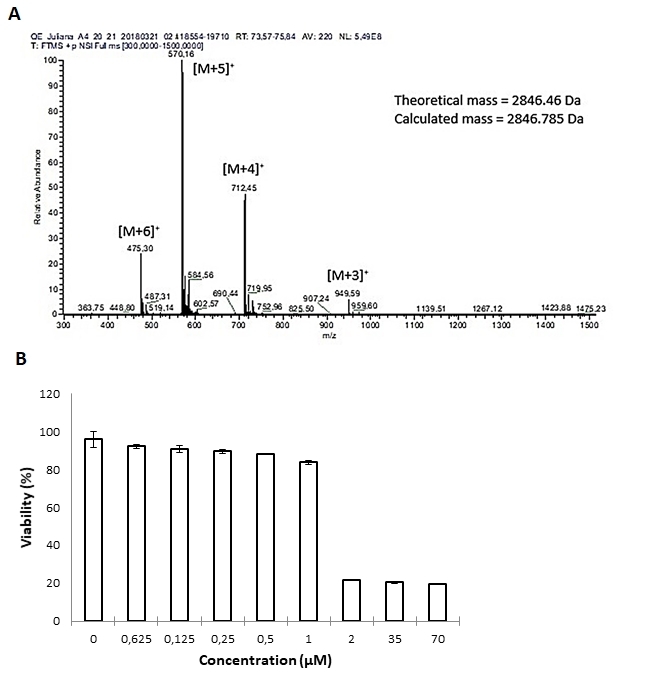
Characterization of melittin used in the CRC treatment. **(A)** Mass spectrometry analysis showing purity and molecular mass of the peptide. **(B)** MTT assay with antiproliferative results of melittin on HT-29/carcinoma colon *in vitro* (mean ± SD). The dose-response relationship demonstrates the effect with increasing dose levels (p < 0.05), which reached its maximum with 2 µM resulting in 80% inhibition*.*

Figure 1A shows the ion envelope with 3, 4, 5, and 6 charges, corresponding to the peptide (2846.78 ± 0.014 Da) before its application in the animals. Besides peptide purity, as no other ions were detected, it was possible to observe the monomeric structure without aggregation. 

The MTT assay showed that melittin inhibited HT-29 proliferation in a dose- and time-dependent manner, decreasing the initial cell number by about 20 % (Figure 1B).

**Figure 2.**
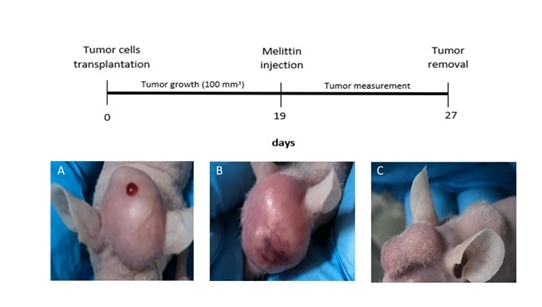
Timeline and CRC bone metastasis progression. **(A)** On day 19: puncture site by melittin intratumoral injection. **(B)** On day 20: inflammatory response one day after melittin. **(C)** On day 27: evident reduction of metastasis size.

When metastasis reached 100 mm^3^ on day 19 (Figure 2A), animals were untreated or treated with melittin. On day 20, the day after melittin injection, local inflammation was observed (Figure 2B), and in the last day of the experiment (day 27) it was possible to see a tumor reduction by visual inspection (Figure 2C) in animals treated with melittin. 

**Figure 3.**
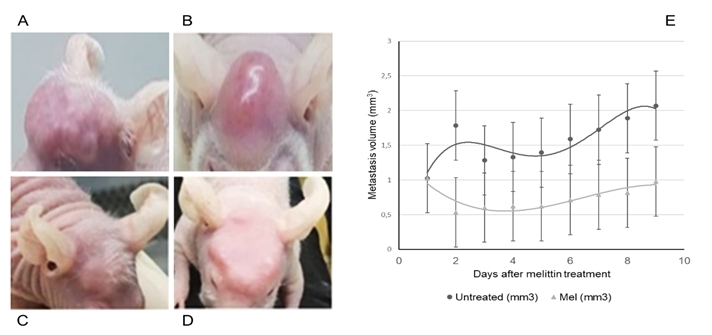
*In vivo* efficacy of melittin. CRC bone metastasis **(A, B)** one day before melittin intrametastatic injection and **(C, D)** seven days after a single dose of melittin (day 27). **(E)** Metastasis normalized volume (mean ± SD) evolution in untreated (n = 3) or melittin-treated animals (n = 6) (p < 0.001). **(C, D)** After intrametastatic melittin injection, bone metastases are smaller than in **(A, B)** untreated animals. Seven days after melittin injection, metastases started growing again.

In order to compare the tumor size of treated and untreated-melittin animals, we determine the volume of the metastasis. As shown in the Figure 3, in untreated animals, a deep, fixed, and vascularized large mass in the cranial region (graft site) was found (Figure 3A and 3B). The macroscopic aspect suggested a carcinoma, showing a single, polypoid mass, homogeneous white tissue, slightly lobulated, with a "fish meat" aspect, fixed deeply in a bone skullcap.

Metastasis reduction was achieved with a single dose of peptide, as shown in the Figure 3C and 3D. Melittin inhibited approximately 50% of the growing metastasis 2 days after the treatment (p < 0.001) (Figure 3E). 

**Figure 4.**
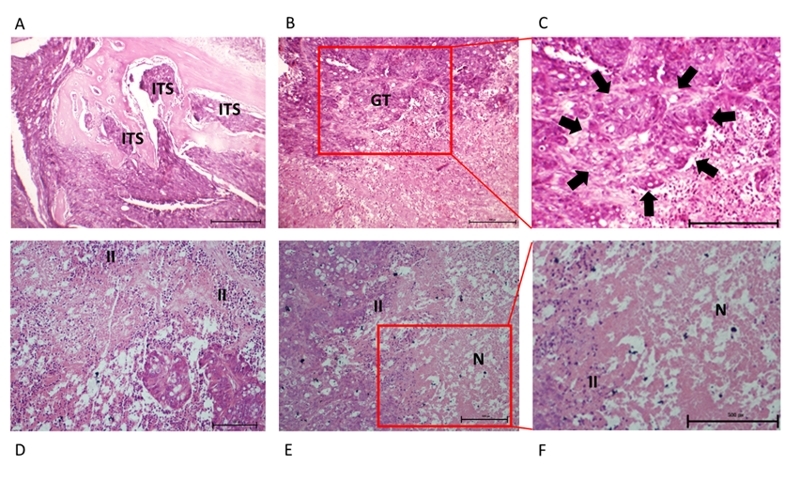
Histopathological characteristics of bone metastasis in colon cancer following inoculation of xenogeneic HT-29 tumor cells **(A, B, C)** in untreated or **(D, E, F)** melittin-treated mice (day 27). **(A B, C)** Metastasis-bearing untreated mice exhibit intense cancer cell invasion into intertrabecular spaces (ITS) characterized by lytic metastasis with bone infiltration, presence of glandular tissue (GT and arrows) and solid sheet areas. **(D, E, F)** Metastasis-bearing melittin-treated mice exhibit inflammatory infiltrated (II) and necrosis (N) areas without cancer cells. Untreated animals (n = 3); melittin-treated animals (n = 6). H&E, **A** [40×], **B**, **D** and **E** [100×], **C** and **F** [400×].

The microscopic analysis of tumor in the untreated animals showed intense cancer cell invasion into intertrabecular spaces, characterized for lytic metastasis with bone infiltration (Figures 4A, 4B and 4C). In melittin-treated animals, microscopy revealed a low mitosis rate. It confirmed the severe inflammatory process, which was resolved in a week after intrametastatic injection. On the last experimental day, a microscopic evaluation showed necrosis and absence of tumor cells in the same area, and metastasis size reduction was observed (Figures 4D, 4E and 4F). 

**Figure 5.**
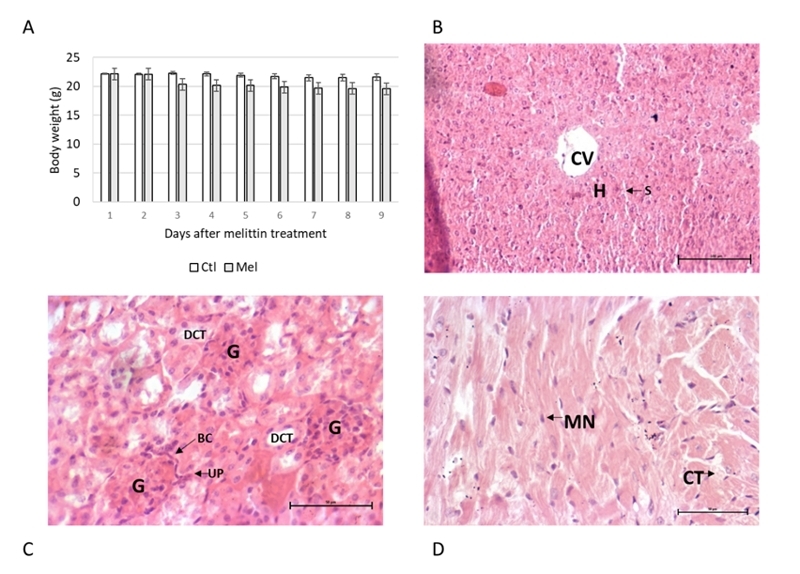
Body weight and histological structure of leaver, kidney and heart following seven days after a single dose of melittin treatment (day 27) showing absence of structural toxicity of melittin. **(A)** Graph showing normalized animal body weight (mean ± SD) over time after the day of melittin injection (day 19) in untreated or melittin-treated animals; weight loss is observed in animals treated with melittin (p < 0.01). Note the stabilization of body weight 24 h after melittin injection. **(B)** Liver micrograph of melittin group showing normal histological structure of the central vein (CV) and surrounding hepatocytes (H) and sinusoids. **(C)** Kidney micrograph of melittin group showing normal histological structure of the glomerulus (G), Bowman’s capsule (BC), urinary polo (UP) and distal convoluted tubule (DCT). **(D)** Cardiac muscle micrograph showing normal histological structure of the striated cardiac muscle, single central nucleus (MN) for each cell and connective tissue (CT) between muscle cells. Untreated animals (n = 3); melittin-treated animals (n = 6). H&E, **B** [200×], **C** and **D** [100×].

The animals treated with melittin did not show signs of envenomation. We have observed that melittin-injected animals had weight loss in the day after the treatment. There is a body weight loss related to the metastasis size (rs = -0.927, p < 0.01). The animals treated with melittin showed greater weight loss than those untreated (p < 0.01) three days after melittin administration (Figure 5A).

However, no signals of toxicity were observed in histopathological analysis of liver (Figure 5B), kidney (Figure 5C) and heart (Figure 5D). The microstructure of the organs was not altered with the melittin treatment.

## Discussion

Bone metastasis affects patients' quality of life and has not been effectively treated. Patients with bone metastases require an active treatment, due to pain, difficulty with ambulation, pathologic fractures, and neurologic deficits. Thus, bone metastasis management deserves new approaches.

Compounds derived from animal venoms are a potential source of therapeutic molecules. Melittin is an amphipathic peptide purified from *Apis mellifera* venom and has shown antitumor effects. In this study melittin was characterized by mass spectrometry to confirm its purity and exact molecular mass and its activity confirmed by MTT, before injection in the bone CRC metastasis animal model. Accordantly, the mass found was 2846,78 Da, already report [26]. 

Melittin shows antiproliferative effect under HT-29/CRC cell. The dose-response relationship demonstrates the effect with increasing dose levels, whose reached its maximum with 2 µM resulting in 80% inhibition (Figure 1B). This antiproliferative effect on colon cell agrees with literature [32,48]. The low tumor cell viability was also demonstrated on gastric [37], lung cancer [49], esophagus [34], lymphoma [29], leukemia [27], ovarian cancer [28], breast cancer [48,50], cervical cancer [43], skin cancers [20,31]. Accordantly, the peptide effects on CRC cells have already been demonstrated [38-40,51]. 

Besides activity on cell cultures, melittin antitumoral activity has also been related in cancer animal models [33,37,41].

Specifically, about metastases, melittin showed activity for lung [49,50], liver [28,51], and esophageal metastasis [34]. For bone CRC metastases, the present data are unprecedented. Here we show that melittin, in a single dose, could reduce the CRC metastasis volume after 7 days. We choose an intratumoral injection in a CRC bone metastasis animal model, to ensure tumor cell death with a safe therapeutic agent for healthy tissues.

On day 19, when metastasis reached 100 mm^3^, animals were untreated or treated with melittin. In untreated animals, a deep, fixed, and vascularized large mass in the cranial region (graft site) was found (Figures 3A and 3B). On day 20, the day after melittin treatment (Figures 2B, 3C and 3D) we observed local inflammation, which was solved in a week after melittin intrametastatic injection. In the following days, a metastases reduction occurs.

The macroscopic aspect suggested a carcinoma, showing a single, polypoid mass, homogeneous white tissue, slightly lobulated, with a "fish meat" aspect, fixed deeply in a bone skullcap. The microscopic sample showed atypical forms of poorly differentiated tumors with poor glandular formation (Figures 4B and 4C), with bone invasion (Figure 4A). After melittin treatment the metastasis showed inflammatory infiltration (Figure 4D), necrosis and absence of tumor cells in the same area (Figures 4E and 4F). 

Accordantly, the ability of melittin to induce necrosis has already been reported [19]. Studies have shown that melittin has cytolytic action by causing phospholipid bilayer rupture, related to necrosis in CRC cells [37-40]. Indeed, after intrametastatic melittin single dose, the metastasis was 53% reduced (Figure 3). 

The intrametastatic strategy improves bioavailability in poor vascularized tissues, such as bone metastasis, simultaneously, prevents biodistribution and, therefore avoids severe adverse effects. In this way, the challenge of the systemic administration of melittin is overcome, as seen in other peptides with linear structures or without disulfide bonds, which can be hydrolyzed by plasma, intra-, and extracellular enzymes.

The intratumoral melittin approach was recently reported, but not using the natural structure of the peptide [20,41]. One study shows the injection of 35 nM modified melittin, able to induce a systemic antitumor response. This optimized peptide was an effective lymph nodes-targeted whole-cell nanovaccine [20]. Another report showed a CRC antitumoral activity of high dose of melittin nanoparticle at 2 mg/kg via intratumoral injection [41]. 

In agreement, in the present study, a high dose was chosen to allow melittin to reach the entire metastatic mass and exert its cytotoxic effect. 

Melittin is known for its hemolytic activity and high toxicity, causing acute renal failure when injected intravenously, with death risk [26]. Although this peptide is significantly more cytotoxic to cancer than health cells [49], representing an advantage to conventional chemotherapeutics, its systemic administration has great impact due to its adverse effects. The clinical use of melittin for cancer therapy is hindered by its notorious side out-target effects. 

To surpass this problem, melittin-conjugates or -derivatives were developed, but involving high costs [20,24,34,35,41,43,52-54]. However, studies describing alternative route of administration are poorly explored so far [31]. 

In the intrametastatic approach, after melittin treatment, animals showed body weight loss, established 48 h after the injection (Figure 5). However, no death or severe toxicity signs were observed here, such as bronchospasm, but some discomfort that could be pain.

The link between the body weight loss and bee venom derivates is rare reported [55]. In the opposite, recent reports showed the increasing in body weight in colitis model treated with melittin [56]. 

Hypothesis regarding weight loss is related to the inflammatory and necrosis process. It was well established that cytokines are involved in the inflammatory process [57,58]. Report showed that the intratumoral injection of α-melittin-NPs resulted in elevated levels of chemokines involved in T and NK cells recruitment and thus, the levels of proinflammatory cytokines such as tumor necrosis factor (TNF)-α, interleukin (IL)-β, IL-1α, and IL-6 were also increased [20]. These pro-inflammatory cytokines regulate the adipocytes proliferation and apoptosis, promote lipolysis, inhibit lipid synthesis, and decrease blood lipids through autocrine and paracrine mechanisms, indicating the formation of a beneficial inflamed tumor microenvironment, although might induce the body weight loss [59]. After 48 h, weight stabilization occurred, probably due to decrease of IL-1β and for TNF-α, which reduced food consumption, in contrast to the effects of IL-1β and TNF-α, IL-6 that do not affect food consumption [60]. 

The absence of systemic effects suggests that melittin did not reach the systemic circulation, even with a high dose injection. The peptide was restricted to the metastasis area when injected into the metastasis' cardinal points. This data was confirmed by the clinical observation and histopathological analysis that revealed no alteration in the structure of the organs and to signals of toxicity. 

The tetramerization of the peptide may have occurred inside the bone metastasis, what explains the local effect of the peptide - due to its size and physico-chemical properties the tetramer could not pass-through membranes and reach the systemic circulation.

Metastasis reduction was achieved with a single dose of melittin, similarly to effect observed with radiation therapy. The peptide inhibited approximately 50% of the growth of metastasis. However, after seven days of a single dose, tumors start growing again (Figure 4D), suggesting that intratumoral injection, once a week, may be suitable for clinical therapy. In a recent paper melittin could be detected in patients up to 30 days after envenomation [61], clearly indicating bioaccumulation corroborating the effects of the single-dose treatment. Besides, melittin has the ability to improves systemic humoral [59,62] and cellular immune response [20]. 

These data provide insight into melittin's effect on tumor growth control and aims to show its use as a therapy promise in bone metastasis. However, it is necessary to explore, in the future, different bone CRC metastasis models to better reflect the systemic process through which cancer cells leave the initial tumor and travel throughout the body to establish a secondary tumor in bone. 

The study concisely presents findings which must be reproducible in future reports. Studies with bigger samples, other cell cancer types and repeated cycles should be carried out to determine if the melittin intrametastatic would lead to complete remission and maintain the low toxicity. Moreover, the intrametastatic melittin combined with drugs or radiotherapy that decrease SREs should bring future perspectives to treat bone metastases with a safe, more effective and less expansive cost.

## Conclusion

We showed for the first time that melittin administration by intrametastatic injection inhibited the growth of bone metastasis in colon cancer. Our data suggest that melittin might be a promising agent for the future development of treatment strategies that seek to reduce bone metastasis skeletal-related impact in CRC patients.
